# Supplementary material for: Shortening of 3′ UTRs in most cell types composing tumor tissues implicates alternative polyadenylation in protein metabolism
Source: RNA. 2021 Dec;27(12):1459–70. doi: 10.1261/rna.078886.121 (PMC8594477; doi:10.1261/rna.078886.121)
Supplement: Supplemental Material [file supp_27_12_1459__DC1.html]

Shortening of 3′ UTRs in most cell types composing tumor tissues implicates alternative polyadenylation in protein metabolism — Supplemental Material 

# Shortening of 3′ UTRs in most cell types composing tumor tissues implicates alternative polyadenylation in protein metabolism

## Supplemental Material

- Supplemental\_Figure\_1.tiff
- Supplemental\_Figure\_2.tiff
- Supplemental\_Figure\_3.tiff
- Supplemental\_Figure\_4.tiff
- Supplemental\_Table\_1.tsv
