# Supplementary figures and images for: Shortening of 3′ UTRs in most cell types composing tumor tissues implicates alternative polyadenylation in protein metabolism
Source: RNA. 2021 Dec;27(12):1459–70. doi: 10.1261/rna.078886.121 (PMC8594477; doi:10.1261/rna.078886.121)

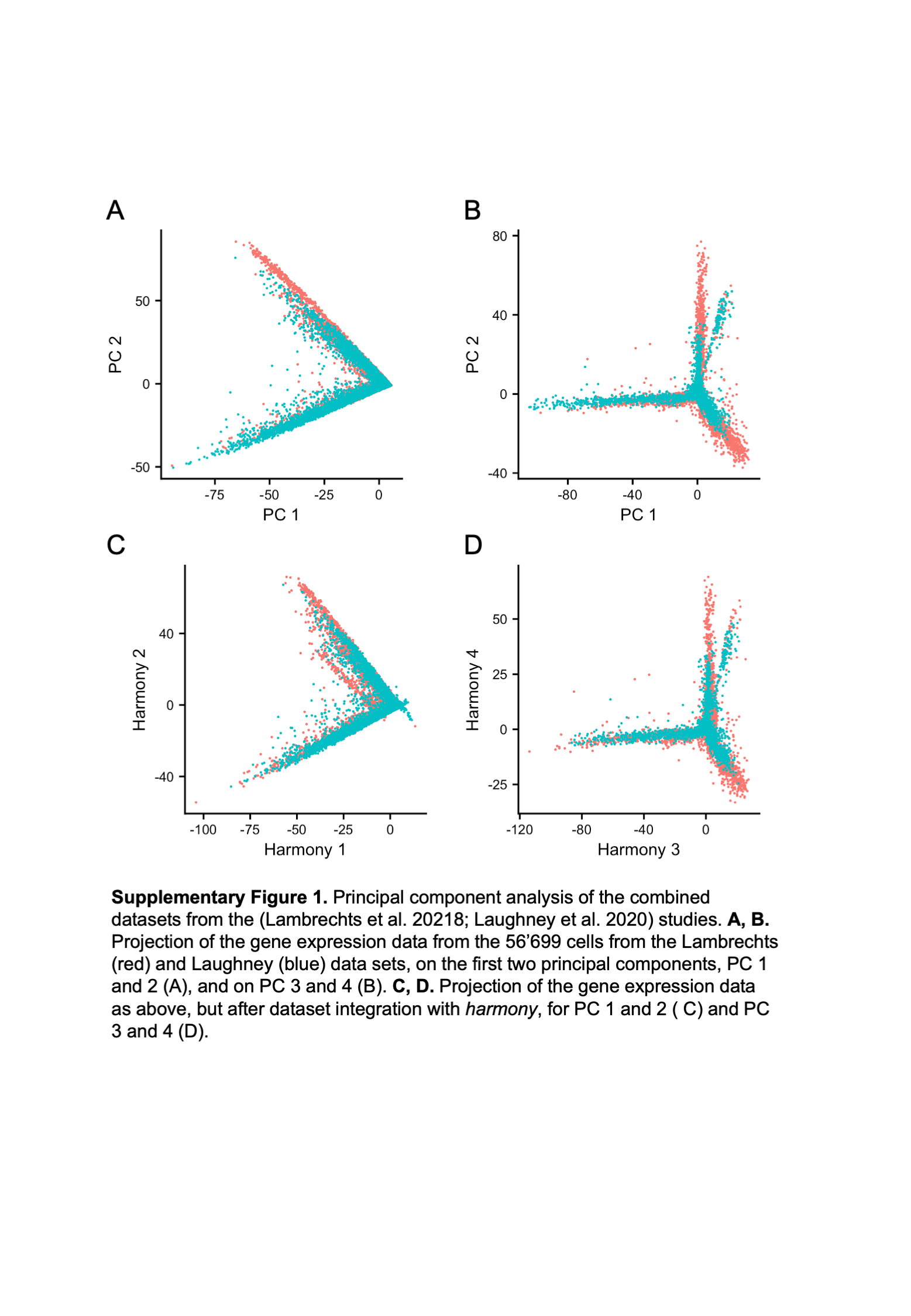

Supplement: Supplemental Material [file supp_078886.121_Supplemental_Figure_1.tif]

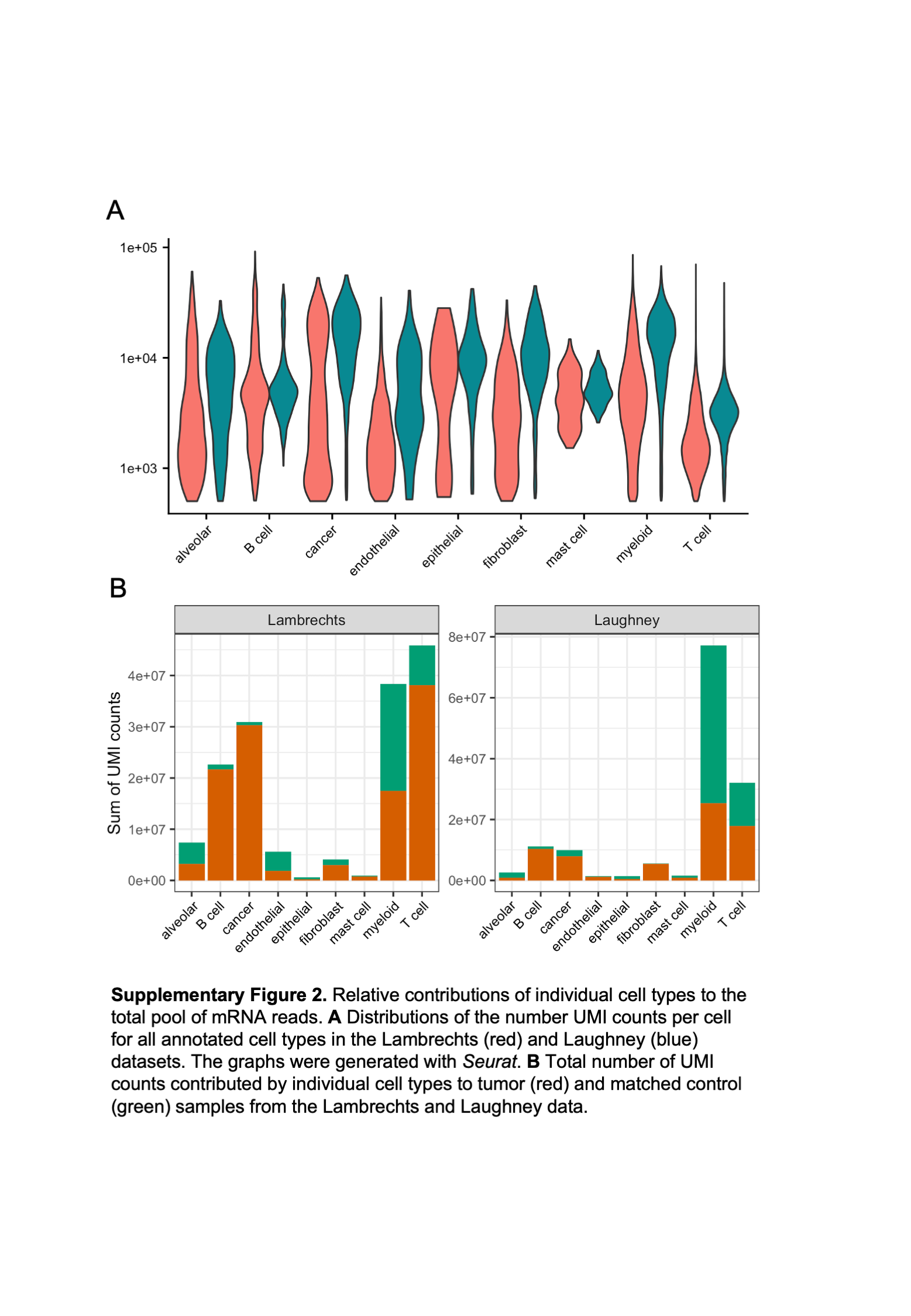

Supplement: Supplemental Material [file supp_078886.121_Supplemental_Figure_2.tif]

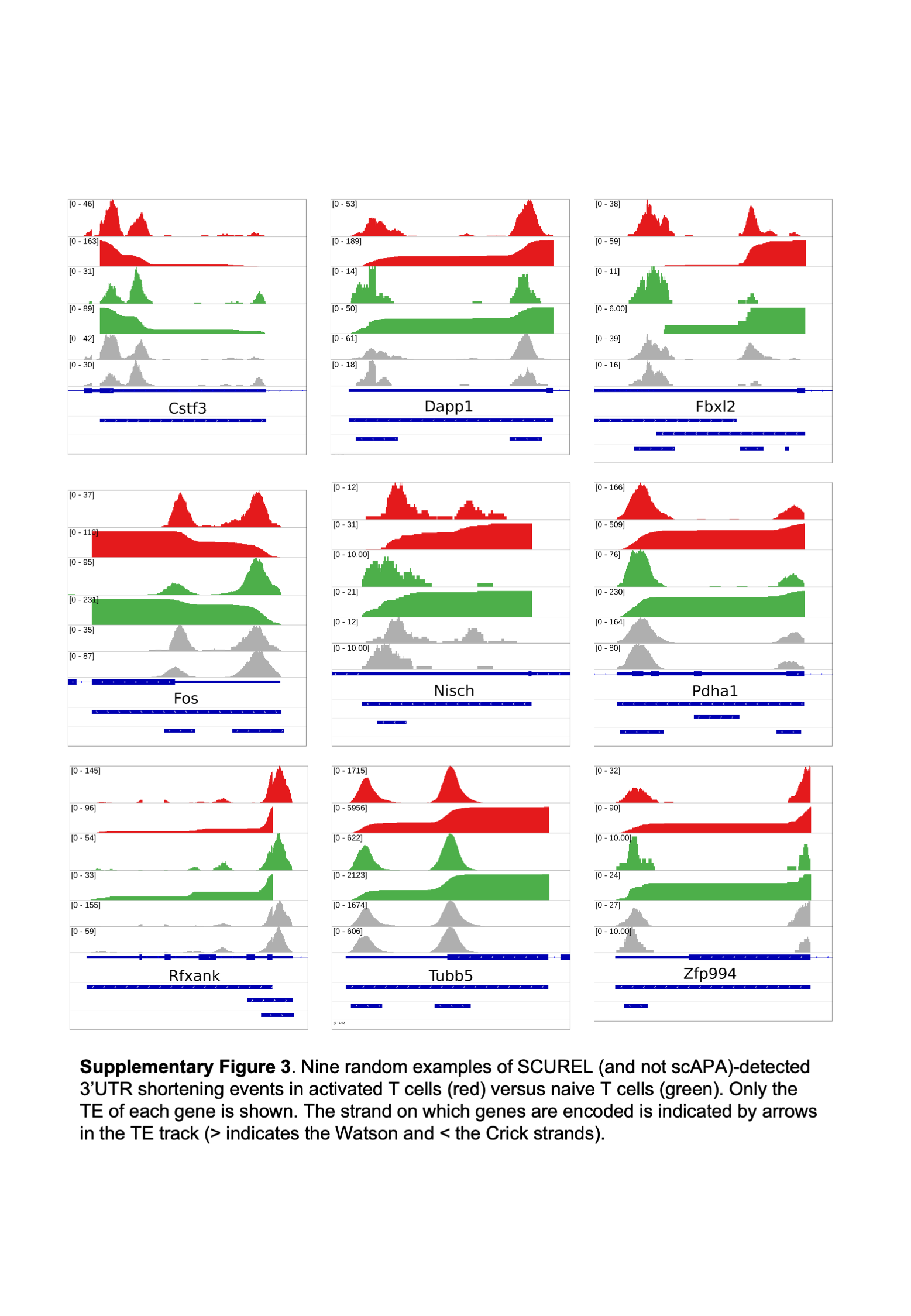

Supplement: Supplemental Material [file supp_078886.121_Supplemental_Figure_3.tif]

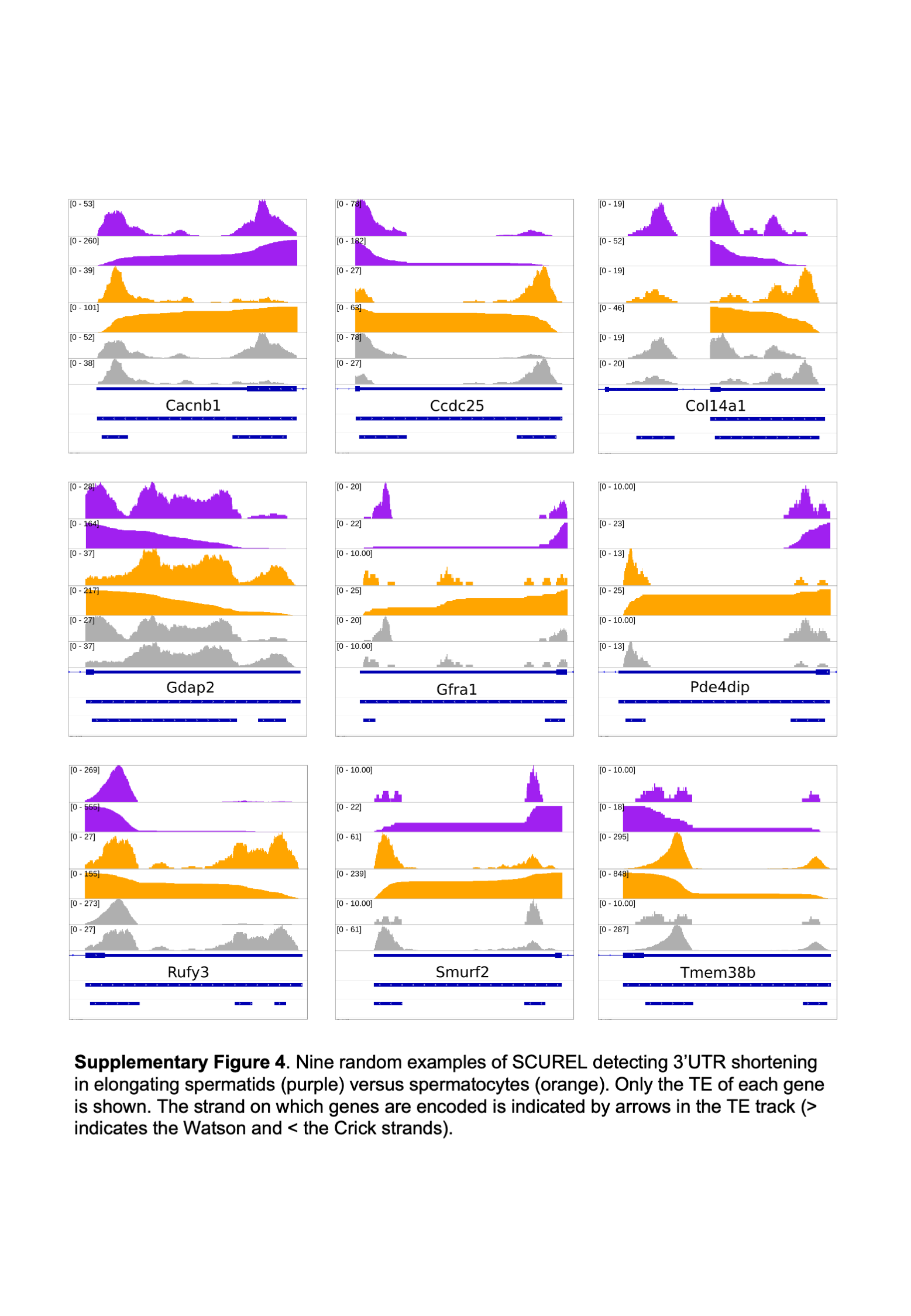

Supplement: Supplemental Material [file supp_078886.121_Supplemental_Figure_4.tif]
